# Supplementary material for: Are carbon emissions trading and green financial instruments synergistic? -Comprehensive quantitative research based on content analysis
Source: PLoS One. 2024 Mar 7;19(3):e0298601. doi: 10.1371/journal.pone.0298601 (PMC10919601; doi:10.1371/journal.pone.0298601)
Supplement: S1 Data — (ZIP) [file pone.0298601.s001.zip › Data/地级市Z_F工作报告.zip!/─┌├╔╣┼/░n═╖╩╨/└┤╫╘╣1⁄2╓┌║┼í╛┬φ┐╦╩2╛▌═°í┐_2018.docx]

各位代表：

现在，我代表市人民政府向大会报告工作，请予审议，并请各位政协委员和其他列席人员提出意见。

一、过去五年工作回顾

市十四届人大一次会议以来的五年，是我市在适应把握引领经济发展新常态中进行深刻调整的五年。在市委的坚强领导下，我们全面落实中央、自治区和市委各项决策部署，全力推动经济社会持续健康发展，向着“提档升级、争创一流”总目标迈出坚实步伐。

——综合实力不断提升。预计全市地区生产总值年均增长7.9%，规模以上工业增加值年均增长9.8%，社会消费品零售总额年均增长9%，固定资产投资年均增长12.8%，一般公共预算收入年均增长9.2%（剔除不可比因素），城乡居民人均可支配收入年均分别增长8%和9%。产业发展后劲持续增强，包钢550万吨稀土钢板材、华云50万吨合金铝、明拓70万吨不锈钢基料等一批重大工业项目相继建成投产。基础设施不断完善，建成高等级公路420公里、旅游公路550公里，完成环城铁路电气化升级改造，生态环保、公共服务和民生社会事业等基础保障能力进一步提升。包银、包西高铁和特高压电力外送通道、千万千瓦级大型现代风电基地、百万千瓦级光伏基地和白彦花煤田开发等一批重大项目获得批准，为今后的发展奠定了坚实基础。

——转型升级步伐加快。全市优质钢、特种钢比重由55%提高到90%，电解铝就地加工转化率由50%提高到80%，战略性新兴产业占规模以上工业比重逐年提高，成为全国工业绿色转型发展试点城市和国家稀土转型升级试点城市。新型商贸、物流、旅游、会展、金融、电子商务等现代服务业蓬勃发展，服务业占地区生产总值比重提高到52%。“菜薯肉乳”四大产业占农牧业比重达到70%，绿色农畜产品加工转化率达到65%，“三品一标”农畜产品数量达到330个。创新发展要素加快集聚，大数据、互联网等新经济、新业态呈现良好发展势头，建成2个国家级重点实验室，创建38个院士工作站，中科院包头稀土研发中心等一批新型研发机构应势而生，各类科研机构累计达到214个，关键共性技术攻关和成果转化项目120项，入选国家科技进步示范市。

——城乡面貌显著改观。规划体系日臻完善，城市空间形态不断优化。举全市之力完成北梁棚户区改造，12.4万北梁居民实现“宜居梦”，其他棚户区改造完成1650万平方米，受益居民28万人，获评全国棚户区改造示范城市。改造老旧小区750个、1877万平方米，受益居民66.3万人，城市直饮水覆盖人口达到198万。新都市区建设全面铺开，城市发展空间布局进一步优化。新建改造城市道路255条，打通断头路114条，“五横八纵”路网基本形成，道路通行能力明显提升。新建改造地下管网1746公里，建成地下综合管廊27.6公里，入选全国首批地下综合管廊试点城市。新增绿地879公顷，1万平方米以上公园广场达到104个，建成区绿化覆盖率达到44.2%。建成百里公交专线和城市绿道，创建景观示范街126条，综合治理街巷104条。城市管理执法体制改革不断深化，智慧城市建设加快实施。美丽乡村建设全面推进，完成危房改造5.2万户，行政村油路通畅率达到100%，48.4万农牧民生产生活条件明显改善。

——生态建设持续加强。京津风沙源治理等国家重点林业工程和大青山绿化、围封禁牧等重点生态工程深入实施，森林覆盖率由15.1%提高到17.6%，草原植被盖度由29%提高到32%。开展重点行业和区域环境综合整治，推进工业节能减排和大气、水、土壤污染防治工程，整治燃煤设施1809台，清理取缔露天煤场307家，搬迁改造高污染企业345户，城区环境质量持续改善。率先推行环境网格化管理，设立环境监管调度指挥中心、环境质量预报预警中心和联合执法队伍，严厉查处生态环境违法行为。加快发展循环经济，国家“城市矿产”示范基地建设扎实推进，工业固废综合利用率达到78%，获评全国生态文明先行示范区、循环经济示范城市、节能减排财政政策综合示范城市。

——改革开放全面深化。经济、行政体制、城镇化、生态、社会事业、对外开放等领域出台改革举措625项，一批重点领域改革取得重大突破。加强生态文明制度建设、完善龙头企业和农牧民利益联结机制、创新同俄蒙合作机制三项先行先试改革扎实推进。国有企业改革深入实施，一机、北重等企业混合所有制改革初见成效。“放管服”力度不断加大，市本级行政审批事项精简59%，审批要件精简21%。“双随机一公开”监管模式全面推开，服务流程进一步优化。商事登记实现“多证合一、一照一码”，全市累计新增各类市场主体12.6万户。非公经济占比达到70%，对全市经济的贡献更加凸显。主动融入国家“一带一路”建设，满都拉口岸实现常年开放，开通中亚国际班列，航空口岸实现临时开放，公路港、铁路港、航空港建设迈出坚实步伐，获批全国商贸物流节点城市，城市知名度和影响力进一步提升。

——人民生活明显改善。民生支出累计达到1510亿元。严格落实精准扶贫政策，全市1.9万贫困人口实现稳定脱贫。就业形势保持稳定，城镇登记失业率控制在3.9%以内。覆盖城乡居民的社会保障体系基本建立，退休人员养老金持续提高，城乡居民医保实现并轨。社会救助体系不断完善，城乡低保标准逐年提高，教育、医疗、住房、取暖等救助工作协调并进，困难群众基本生活得到有效保障。教育事业全面发展，教育教学水平稳居全区前列。医药卫生体制改革全面推进，获批全国首批健康城市试点。文化事业和文化产业繁荣发展，成功创建国家公共文化服务体系示范区。公共体育服务体系不断健全，获评“中国十佳运动休闲城市”。地震气象人防、档案史志社科、外事侨务贸促和工青妇、儿童老龄、残疾人、红十字等各项事业都取得了新进展。安全生产、食品药品安全工作不断加强，平安包头建设深入推进，拥军爱民、民族团结、宗教和顺的和谐稳定局面不断巩固，继续保持了社会治安综合治理“长安杯”、“全国双拥模范城”、“全国法治宣传教育先进城市”和“全国文明城市”等殊荣。

——政府自身建设不断加强。坚决维护以习近平同志为核心的党中央权威和集中统一领导，认真落实全面从严治党要求。深入开展党的群众路线教育实践活动、“三严三实”专题教育和“两学一做”学习教育，认真贯彻中央八项规定、国务院“约法三章”精神，严格落实自治区、市委配套规定，严控办公用房标准，完成公务用车制度改革，政风明显好转。认真落实市人大及其常委会的决议和决定，主动接受人大法律监督和工作监督。自觉接受政协民主监督，主动听取各民主党派、工商联、无党派人士和专家学者意见建议。全面推进依法行政，健全完善政府议事决策规则、“三重一大”集体决策、行政决策合法性审查和政府法律顾问等制度，加大审计、监察力度，大力推行政务公开，廉洁政府建设不断加强。

各位代表，刚刚过去的2017年，我们隆重庆祝了自治区成立70周年，在努力保持经济平稳发展的基础上，以更大的决心、更大的力度从根本上转变发展理念、转变发展方式。针对中央巡视“回头看”和自治区党委巡视指出的问题，我们主动认领、坚决整改，及时停建压缩了地铁等55个政府性投资项目，压减投资702亿元；对主要经济指标作了调整，目的是压实总量、提高质量，卸下包袱、轻装上阵。预计完成地区生产总值3450亿元，按可比口径增长6%；固定资产投资3010亿元，增长2%；社会消费品零售总额1500亿元，增长7%；一般公共预算收入137.6亿元，下降49.3%，剔除虚增空转因素后同比增长6.1%；城乡居民人均可支配收入分别达到44230元和15870元，均增长8%，完成了调整后的预期目标。用电量、货运量等先行指标保持两位数增长，主要工业品量价齐升，规模以上工业企业利润大幅增长，就业比较充分，经济运行总体稳中有进、稳中向好。一年来，我们重点抓了以下工作：

一是全力以赴稳运行。主动应对经济下行压力，强化经济运行研判调度，实施重点企业市级领导包联帮扶责任制，不断完善国有企业改革发展政策体系，积极帮助企业解决运行中的实际困难，包钢扭转了连续3年亏损局面。积极争取自治区电力多边交易、基本电费减免政策，为企业降低用电成本19.4亿元。制定出台扶持“百年老店”发展的政策，授予26家企业“百年老店”称号。强化跟踪督办推进机制，全力推动亿元以上重大项目建设，完成投资1924.6亿元。实施招商引资项目1120个，引进国内（区外）资金到位879.2亿元。落实供给侧结构性改革任务，淘汰48万吨落后钢铁产能，化解60万吨煤炭过剩产能。推动棚户区改造货币化安置，新建住房库存面积较2016年底下降5%。积极稳妥推进政府债务化解，金融机构不良贷款率控制在合理区间。取消停征行政事业性收费和基金16项，为企业减轻负担9235万元。

二是坚持不懈调结构。牢牢把握提质量、增效益的发展方向，推进传统产业优化升级，新增稀土高强度防护用钢、特种越野车用钢等一批新产品，北方股份110吨级电动轮矿用车成功下线，包头盛泰铝轮毂、汇众汽车摇臂项目建成投产，联网数控机床达到602台（套）。发展壮大战略性新兴产业，自治区级稀土新材料产业园区挂牌成立，阿特斯光伏一期等新能源项目建成投产，启动建设全国首个军民融合综合评估中心、中国技术交易所包头中心、自治区军民融合大数据中心。全面提升科技创新能力，全社会研究与试验发展经费（R&D）占地区生产总值比重达到1.8%，组建信息技术大数据研究院等7所研发机构，国内首台磁共振医疗车研发成功，一机集团自主研发的4×4防雷车填补了该领域空白，世界首条隧道窑绿色环保稀土着色剂中试线投入使用，进入首批全国中小企业知识产权战略试点城市行列。大力发展现代服务业，成功举办第18届中国绿色食品博览会、中国特色旅游商品博览会等62次会展活动。包头海洋馆等旅游项目建成运行，旅游总收入492.2亿元，增长23.5%。深化农牧业供给侧结构性改革，绿色农畜产品进社区工程和“七个一流”现代农牧业体系建设有序推进，粮食安全盟市长责任制考核居自治区首位，成为全国绿色食品溯源试点城市。

三是坚定不移增活力。着眼于破除体制机制障碍，协同推进“放管服”改革，实行行政权力动态管理和清单管理，推行“互联网+政务服务”模式，市本级387项公共服务事项全部实现网上办理，市政务大厅荣获全国“百优大厅”称号。实行公共资源交易目录制度，完成市县交易平台整合，工程建设交易实现全流程电子化。稳步推进国有企业剥离办社会工作，包钢医院、包钢职业技术学院等机构实现委托管理，“三供一业”移交工作全面启动。36户市属企业纳入国有资产集中统一监管体系。实施“六个一”提升工程，82%的龙头企业与农牧民形成有效利益联结。全面推进国有林场、土地确权和不动产统一登记制度改革，草原确权基本完成。加快发展外向型经济，开工建设国家进口肉类指定口岸、中蒙互市贸易区，B型保税物流中心获准建设，满都拉口岸过货量突破200万吨。完成包头国际机场航站楼改造，机场旅客吞吐量突破200万人次，进入国内中型机场行列。

四是建管结合抓城建。立足完善功能、提升品质，强化城乡规划战略引领和刚性控制，城市总体规划（2011—2020）获得国务院批准，入选全国第一批城市设计试点和全国“城市双修”试点。北梁腾空区首批重点项目全面启动，新都市区开发建设扎实推进。110国道改造、沼南大道等重点道路和综合管廊项目加快实施，军工文化主题公园、奥林匹克公园全面开放，“两馆一中心”基本建成，劳动公园、八一公园等老旧公园完成改造，赛汗塔拉新增规划绿地面积2800亩，成为名副其实的万亩城中草原。大力发展装配式建筑，加快提升工程建设品质，获评国家装配式建筑示范城市。持续加强城市管理，实施城市环境综合整治“五化”工程，顺利通过“国家卫生城市”复审。

五是多措并举优生态。始终坚持绿色发展理念，大力开展生态保护修复，实施国家林业重点工程40万亩、重点区域绿化3.7万亩。积极推进大青山南坡生态综合治理工程，完成绿化修复6.4万亩，清理整顿违法企业项目256个，自然保护区工矿企业全部退出。开展废弃矿井专项整治，累计封堵废弃矿井643个。重拳治理环境污染，实施污染治理工程130项，划定300平方公里高污染燃料禁燃区和192处畜禽禁养区，完成原煤散烧整治9.5万户，全市空气质量优良天数达到277天，比上年增加8天，全面完成中央环保督察反馈意见年度整改任务。实施城市水生态综合治理工程，完成30公里河道治理、污水控制、水景观建设等改造任务，小白河等湿地保护开发项目加快推进，“河长制”得到全面推行。

六是用心用情惠民生。全面贯彻以人民为中心的发展思想，投入扶贫资金6.5亿元，实施精准脱贫攻坚，建立贫困学生交通补贴、免费免试接受职业教育、医疗扶贫救助金制度，开展农畜产品认购、园林绿化、义拍捐赠等社会公益扶贫模式，扶贫质量进一步提高。着力推动创业创新，建设和认定市级以上创业园孵化基地73个，7218名就业困难人员实现再就业，为1.1万名创业者发放创业担保贷款10亿元，居全区首位。高度关注弱势群体，全力开展农牧民工工资清欠工作，投入2.4亿元提高养老、医疗、低保等7项社会保障和救助标准。开工建设综合老年养护院，加快推进儿童福利院、老年福利院、救助管理站等工程建设。新建18所中小学、幼儿园，投入1.7亿元实施高中分层教学等三项制度改革，贫困学生资助政策实现全覆盖。积极推进市儿童医院等项目建设，与国内知名医疗机构合作共建院士工作站8个，投入3000多万元为年满50周岁农牧民实行免费体检，为偏远乡镇卫生院配备流动卫生服务车和医疗设备。组织实施原创剧目展演、群众文化艺术等一系列公共文化活动，成功举办中国体育文化博览会、中国体育旅游博览会和首届草原国际足球节等重大活动。深入实施“七五”普法规划。建立健全社会治安防控体系，启动推行“三见警”常态化巡逻机制，持续开展缉枪治爆、打击“黄赌毒”等专项行动，深入开展安全生产大检查和信访积案化解“百日攻坚”，治安和刑事案件、安全生产事故整体下降，“平安包头”建设成果持续巩固。

过去一年，我们进一步加强政府自身建设，深入推进依法行政。牢固树立“四个意识”，不断强化责任担当，坚决整改中央巡视“回头看”、自治区党委巡视和市委巡察指出的问题。高度重视人大代表建议和政协委员提案办理工作，共办理人大代表建议和政协委员提案427件，办结率100%。严格落实党风廉政建设责任，全面开展政府系统“作风转变年”活动，持续开展重大政策落实、财政民生资金、领导干部经济责任审计，强化行政监察和督查问效，执行力落实力进一步增强。更加自觉地接受社会监督，完善政务公开和新闻发布制度，全年举办新闻发布会48次。严控“三公”经费支出，腾出更多资金用于保障和改善民生。

各位代表！五年的发展成就来之不易。这是习近平新时代中国特色社会主义思想科学指引的结果，是市委统揽全局、坚强领导的结果，是市人大、市政协认真监督、大力支持的结果，是全市各级干部群众同心同德、苦干实干的结果，是社会各界关心关注、共同努力的结果。在此，我代表市人民政府，向全市各族人民，向所有为包头建设发展作出贡献的同志们、朋友们表示衷心的感谢、致以崇高的敬意！

各位代表！五年的奋斗历程，让我们深刻体会到：推动包头的改革发展事业，必须坚持党的领导。党的领导是各项事业成功的根本保障。我们只有牢固树立“四个意识”，坚决维护以习近平同志为核心的党中央权威和集中统一领导，全面贯彻落实中央、自治区和市委的决策部署，才能确保改革发展各项事业始终沿着正确的方向前行。必须坚持工业强市。工业是包头的立市之本，更是强市之基。我们只有坚决贯彻落实新发展理念，把转变经济发展方式的重中之重放在工业提档升级上，强化科技创新，深化改革开放，才能破解发展难题，厚植发展优势，实现更有质量、更有效益、更可持续的发展。必须坚持生态优先。绿水青山就是金山银山。我们只有坚定不移地走绿色发展之路，形成节约资源和保护环境的空间格局、产业结构、生产方式、生活方式，才能实现人与自然的和谐共生，建成美丽家园。必须坚持依靠群众力量。发展为了人民，发展依靠人民。我们只有坚持以人民为中心的发展思想，从解决人民群众普遍关心的突出问题入手，不断满足人民群众日益增长的美好生活需要，充分激发人民群众的积极性主动性创造性，才能凝聚起建设包头的强大正能量。必须坚持艰苦奋斗的创业精神。艰难困苦，玉汝于成。包头的发展历程炼就了艰苦奋斗的创业精神。我们只有不断传承和发扬这种精神，敢于攻坚克难、勇于开拓创新，实事求是、扎实苦干，才能肩负起新时代赋予的新使命。

各位代表！站在新的历史起点，我们也清醒地认识到，发展中还面临着不少困难和问题。主要是：产业支撑能力不强，传统支柱产业仍处于价值链中低端，新兴产业尚未形成规模，非公经济发展还不充分，缺乏带动作用强、对经济贡献率大的项目；地区综合优势未能有效发挥，龙头企业带动作用不明显，驻市企业与地方经济未能形成深度融合，优势产业集群尚未形成；创新能力不强，科技成果转化率不高，新技术、新业态、新经济开发和培育滞后，经济转型升级压力大；税收增长乏力，财政收支矛盾凸显，化解政府债务任务艰巨；城乡发展不平衡问题依然突出，基础设施还不完善，生态环境比较脆弱，空气质量亟待改善；民生和社会事业领域的短板尚待补齐，人民群众在就业、教育、医疗、安居、养老等方面还面临不少难题；部分干部墨守成规、不敢担当，一些单位作风漂浮、工作不实，与新时代新任务新要求还不相适应。问题导向就是努力方向，我们将切实采取有力措施，认真加以解决。

二、今后五年的总体思路

今后五年，是全面建成小康社会的决胜期，也是实现“两个一百年”奋斗目标的历史交汇期。党的十九大吹响了决胜全面建成小康社会、开启全面建设社会主义现代化国家新征程的集结号。新一届政府要切实增强责任感和使命感，不忘初心、牢记使命，埋头苦干、锐意进取，不断开创改革发展事业和现代化建设新局面。

政府工作的总体要求是：全面贯彻党的十九大精神，以习近平新时代中国特色社会主义思想为指导，统筹推进“五位一体”总体布局，协调推进“四个全面”战略布局，坚持稳中求进工作总基调，坚持新发展理念，紧扣社会主要矛盾变化，按照高质量发展要求，认真落实中央、自治区、市委的决策部署，紧紧抓住发展这个解决一切问题的基础和关键，突出抓重点、补短板、强弱项，坚决打好防范化解重大风险、精准脱贫、污染防治攻坚战，坚决守好发展、民生、生态三条底线，进一步增强发展后劲，进一步提升城市品位，进一步改善人民生活，进一步优化生态环境，推动各项事业提档升级、争创一流，确保与全国同步建成经济更加发展、民主更加健全、科教更加进步、文化更加繁荣、社会更加和谐、人民生活更加殷实的全面小康社会，全面开启社会主义现代化建设新征程，奋力谱写“建设亮丽内蒙古，共圆伟大中国梦”的包头篇章。

按照总体要求，重点要抓好六个方面的工作：

——更加注重增强发展后劲，努力打造我国西部地区最具竞争力的现代工业城市。深化供给侧结构性改革，提高供给体系质量，优化各类要素配置，大力振兴实体经济。强化一盘棋意识，树立“大包头”理念，打破所有制和隶属关系界限，挖掘整合辖区内的一切生产要素，推动驻市企业与地方经济深度融合，加快构筑以央企区企等优势企业为龙头、众多中小企业协同发展的产业集群。以创建“中国制造2025”示范区为契机，运用互联网、大数据、新技术等手段改造提升传统产业，推动钢铁、铝业、装备制造等产业向高端化、高技术化、集群化方向发展。大力发展战略性新兴产业和现代服务业，在中高端消费、绿色低碳、共享经济等领域培育新增长点，形成新旧动能协调拉动的经济发展格局。发挥稀土资源的绝对优势，以国家级稀土新材料产业园区为载体，强化顶层设计和政策配套，加强科技研发和成果转化，提高白云鄂博矿产资源综合开发利用水平，拓展稀土开发应用的广度和深度，打造“世界稀土产业之都”。

——更加注重科技创新驱动，加快建设科技创新高地和创新型城市。构建形成以创新为主要引领和支撑的经济体系和发展模式，建设国家可持续发展议程创新示范区。围绕企业技术改造、产品开发和产业发展需求，实施高校创新能力、科研机构研究能力提升计划，加快建设稀土功能材料创新中心等一批国家级科技创新平台。依托龙头企业、国家重点实验室、院士工作站和各类科研院所，针对重点领域、关键环节开展一批科技集中攻关和中试研究，促进产学研深度融合和科技成果落地转化，让创新链更好地服务产业链。积极推动军民融合深度发展，构建全要素、多领域、高效益的军民融合发展格局。深入实施人才强市战略，优化人才培养引进政策和服务体系，引进一批领军人才，集聚一批高技能人才，培育一批“包头工匠”，加快构建科技创新能力体系。

——更加注重深化改革开放，着力建设更具活力动力的开放型城市。全力抓好习近平总书记嘱托内蒙古先行先试的三项改革任务落实，深入推进经济、生态、行政体制等重点领域改革攻坚，进一步破解体制机制障碍。深化“放管服”改革，充分激发民间投资活力动力，大力发展非公有制经济，使包头成为吸引投资、创新创业的热土。深化金融体制改革，健全金融监管体系，积极化解政府债务，坚决打好防范化解重大风险攻坚战。深化国企国资改革，完成剥离国有企业办社会职能工作，全力支持包钢、包铝、一机、北重、北奔、二O二、二冶等企业做强做优做大。加快口岸、外贸、外资、跨境电商等平台建设，鼓励企业通过产能合作开拓国际市场，构建全方位对外开放新格局。积极落实呼包鄂协同发展战略，推进各领域互联互通，提升城市辐射力带动力。

——更加注重提升城市品位，倾心打造以人为核心的宜居宜业现代化城市。坚持人民城市为人民，用科学规划引领城市发展，划定生态保护红线、永久基本农田、城镇开发边界三条控制线。实施“功能完善、景观提升、交通优化、文旅融合”四大工程，构建系统完善、运行高效的现代化城市基础设施体系。不断强化新都市区基础设施、公共服务和综合配套设施，建设贯通东西、连接南北的城市新中心。健全城市管理体制机制，加快“智慧城市”建设，提高标准化、精细化、智能化、人性化管理水平。大力实施乡村振兴战略，坚持农牧业和农村牧区优先发展，建立健全城乡融合发展的体制机制和政策体系，着力优化“三带六区”农牧业产业布局，加快推进农牧业农牧区现代化。践行社会主义核心价值观，加强城乡精神文明建设和全社会诚信体系建设，全面提升城乡内涵品质。

——更加注重优化生态环境，奋力建设人与自然和谐共生的美丽包头。坚决打赢污染防治攻坚战，实行最严格的生态环境保护制度，开展“山水治理、大气防控、水土改善”三大行动，确保主要污染物排放总量大幅减少，城区环境质量明显改善。开展大规模国土绿化行动，实施好天然林保护、退耕还林等国家重点工程，巩固好围封禁牧成果。加强生态保护修复，统筹山水林田湖草系统治理，抓好大青山南坡地质环境治理及生态恢复。大力发展绿色低碳产业，培育壮大节能环保产业，推进资源全面节约和循环利用，强化节能减排，建立健全绿色低碳循环的经济体系，推动生态环境质量大幅改善。

——更加注重改善人民生活，全面提升全市人民的获得感、幸福感和安全感。强化精准扶贫、精准脱贫举措，坚决打赢精准脱贫攻坚战。坚持以产业、创业带动就业，落实全民参保计划，不断提升城乡居民收入和社会保障水平。推进教育强市建设，优化资源配置，提高教育质量，促进教育公平，打造全区一流、人民满意的教育。深化医药卫生体制改革，全面建立基本医疗卫生制度、医疗保障制度和优质高效的医疗卫生服务体系，积极发展多形式、多层次的城乡养老事业，加快建设健康城市。完善公共文化服务体系，大力弘扬乌兰牧骑优良传统，实施文化惠民工程。坚决抓好安全生产、食品药品安全、社会治安等工作，确保社会大局和谐稳定。

各位代表，新时代赋予新使命，新时代要有新作为。包头有六十多年的工业基础，有相对完备的产业体系，有较为雄厚的科研和人才优势，有健全完善的城市功能，有蝉联五届文明城市的深厚底蕴，有艰苦奋斗的创业精神，有深深热爱着这座城市的286万勤劳智慧的各族人民，特别是在党的十九大精神鼓舞下，市委团结带领全市广大干部群众凝聚形成了干事创业的强大合力，我们完全有基础、有条件、有信心、有能力实现既定目标。只要我们戮力同心、埋头苦干，就一定能够挺钢铁脊梁，振包头雄风!

三、2018年工作建议

2018年是贯彻党的十九大精神开局之年，是改革开放40周年，是决胜全面建成小康社会、实施“十三五”规划承上启下的关键一年，我们要认真贯彻落实中央经济工作会议、自治区党委十届五次全会暨全区经济工作会议、市委十二届四次全会精神，统筹做好经济社会发展各项工作。综合考虑当前和长远、需要和可能、速度和质量等各方面因素，建议主要预期目标为：地区生产总值增长7%左右，固定资产投资增长8%左右，社会消费品零售总额增长8%左右，一般公共预算收入增长6%左右，城乡常住居民人均可支配收入均增长7.5%左右，城镇登记失业率控制在3.9%以内，政府债务化解完成年度任务，全面完成国家和自治区下达的各项约束性指标。

实现上述目标，要重点抓好七个方面的工作：

（一）深化供给侧结构性改革，全力振兴实体经济。坚持把发展经济的着力点放在实体经济上，把提高供给体系质量作为主攻方向，坚定不移推动经济发展质量变革、效率变革、动力变革。

一是深入挖掘内在潜力。全力推进企地融合，制定出台支持实体经济、促进企地融合发展的政策措施，鼓励支持中小企业围绕央企区企等优势企业开展产业协作配套、科技成果转化和共享经济服务，策划实施企地融合项目30个以上。建立企地交流合作运行机制和绿色通道，搭建互动交流、务实管用的合作平台，最大限度地发挥出全市经济发展的综合优势。支持实体企业成长，强化煤电油气运等生产要素保障，综合施策降低企业融资、物流、用能成本和税费负担，帮助企业解决实际困难，充分释放有效产能。健全完善中小企业公共服务平台功能，降低初创期中小微企业经营成本，培育“专精特新”中小企业150户。扩大“助保金贷款”覆盖面和资金池，惠及企业100户以上，发放助保金贷款5亿元以上。落实好关于非公经济发展的一系列政策措施，继续清理规范涉企收费，着力优化实体经济发展环境。实施企业家培育工程，依法保护民营企业合法权益，构建亲清新型政商关系，营造利于企业发展的良好氛围，让企业家安心创业、安心发展。大力弘扬工匠精神，开展质量提升行动，推动标准化建设，加强商标使用、管理和保护，打造一批知名“百年老店”，让更多的包头品牌出去“闯天下”。

二是实施精准有效投资。充分发挥投资对优化供给结构的关键性作用，按照提高质量、优化结构、产业集聚、注重效益的原则，毫不放松地抓招商、抓项目，年内组织实施亿元以上重点项目680个，完成投资1500亿元以上。加强产业项目建设，鼓励支持现有企业实施产业升级项目，开工建设晶澳太阳能单晶硅、盛泰50万只卡巴轮等130个项目，加快推进神华二期、神雾化工等项目建设，力争北方嘉瑞高强度聚乙烯、丰川高压化成箔生产线等300多个项目竣工。加强基础设施建设，加快实施东河至白云鄂博、110国道北绕城等公路项目，确保满都拉口岸至白云鄂博、东河至托县等公路建成通车。积极推动包银高铁开工建设，加速融入国家高速铁路网。力争石拐、达茂通用机场建成投入使用，争取包头机场升级为国际机场。实施招商引资“市长旗县区长工程”，统筹整合全市各类招商资源，加强招商队伍建设，发挥产业体系和城市配套的综合优势，创新招商方式，注重以商招商、精准招商，全方位对接国际国内龙头企业，力争在稀土新材料、高端装备及智能制造、总部经济、新型服务业等领域引进一批大项目好项目，年内引进国内（区外）资金增长8%左右。

三是强化科技创新驱动。突出企业创新主体地位，强化知识产权保护和运用，引导企业加大研发投入，重点培育50家国家高新技术企业、10家科技小巨人企业。支持新型研发机构建设，鼓励重点科研院所和企业申报国家、自治区级科技企业孵化器、重点实验室、企业技术中心和工程技术中心，组织实施重大科技专项30项以上。推动科技成果转化，充分发挥院士工作站等研发机构作用，建立以市场为导向、产学研深度融合的技术创新体系，推动创新要素向产业和企业集聚，支持绿色环保稀土着色剂、伺服电机等科技成果产业化。抓好军民融合，鼓励军转民、民参军，培育认定10家军民融合科技创新示范企业，组织实施15项以上军民协同创新重点项目，全力创建国家军民融合创新示范区。强化人才支撑，探索建立“人才+项目+资本”协同引才模式，深入实施高技能人才“聚匠工程”和“国际认证引育工程”，开展享受政府特殊津贴评选和青年创新人才项目资助活动，力争培养引进高层次人才1000人左右。

四是强化金融服务保障。支持金融机构回归本源，打通金融服务实体经济的“血脉”。创新服务模式，积极推动组建金融租赁公司，加强与外埠非银行金融机构的合作，帮助和支持企业拓宽融资渠道、降低融资成本。依托多层次资本市场，支持金海新能源、威丰电磁等企业在主板、创业板上市，鼓励更多企业在新三板、区域股权市场挂牌，支持包钢等企业推进市场化法治化债转股，提高直接融资比重。持续优化金融环境，树立地区良好形象，合理运作城市资源，吸引更多资金助力基础设施建设。大力发展绿色金融和普惠金融，加大金融对节能环保、精准脱贫、“三农三牧”支持力度。促进科技与金融紧密结合，与中科院包头稀土研发中心、上海交大包头材料研究院及有关投资机构合作设立风险投资基金，加快构建科技金融服务链。

（二）全力推进产业转型升级，加快构建现代产业体系。深入落实“五个结合”，着力做强做优存量，做大做特增量，以工业提档升级引领产业全面升级。

一是全力振兴工业经济。改造提升传统产业，制定出台传统工业优化升级三年行动方案，重点在增品种、提品质、延链条上下功夫，实施明拓160万吨铁素体不锈钢、威丰冷轧取向硅钢三期、固阳50万吨轻金属材料等重大产业项目，培育发展高品质特殊钢、新型轻合金制造等产业。加大传统机床数字化改造力度，扩大网络协同制造平台的应用领域，促进信息技术在企业研发设计、生产制造、经营管理、销售服务等全流程和全产业链的集成应用。大力发展战略性新兴产业，加快推进通威高纯晶硅、亚洲硅业超纯硅和阿特拉斯切片及电池组件等项目建设，打造投资200亿元的晶硅—切片—光伏组件—太阳能电站产业链。实施宝钢化工针状焦、杉杉负极材料、北京双杰电池隔膜及昊明新能源电池等项目，打造投资150亿元的煤焦油—针状焦—负极材料—电池—新能源汽车全产业链。制定落实智能制造三年行动计划，实施医疗器械、伺服电机、工业机器人、智能车间等现代装备制造项目，推动组建区域网络协同制造联盟，建设智能制造创新体验中心，加快构筑智能制造互联网生态圈和制造业共享经济圈。积极发展大数据产业，重点完善基础数据库建设和无线城市应用，建设高速宽带信息通信网，加快“智慧包头”建设。

全力做强做优做大稀土产业，积极争取国家和自治区支持，制定支持稀土产业发展专项政策，以独特的资源优势和宽松的政策环境，大力引进国内外具有竞争实力的研发和应用企业。建设国家稀土功能材料创新中心，整合稀土科研院所、企业创新资源，提高稀土、铌、钍等资源开发应用水平，扩大稀土功能材料和终端应用产品比重，培育新的经济增长极。大力实施园区振兴计划，优化园区布局和产业定位，健全园区管理体制和工作机制，科学完善基础设施，提高承载服务能力。建立健全“僵尸企业”退出处置机制，推动化解过剩产能，破除无效供给，盘活土地等存量资源。提高投资强度和产出效益，促进园区集约集聚发展。

二是加快发展现代服务业。制定出台支持现代服务业发展的实施意见，筹备召开全市现代服务业发展大会，推进现代服务业提质增效。促进生产性服务业向专业化和价值链高端延伸，着力扩大钢铁、有色、建材等物流规模，发展绿色物流、冷链物流、城乡配送等新业态，支持物流企业拓展仓储、金融等增值服务，推动物流业标准化改造，提高物流全链条服务时效。大力发展互联网共享经济等新经济、新业态、新商业模式，加快乐享机械营销网、诚昊启元无车承运人互联网平台+冷链物流储配送项目建设。依托国家工程实验室包头信息技术大数据研究院，加快培育壮大稀土、钢铁、煤炭电商交易平台，推进苏宁内蒙古结算中心建设，进一步扩大电子商务交易规模。支持会展产业市场化发展，举办各类大型展会60场以上。推动生活性服务业向精细化和高品质转变，高标准实施中东奥特莱斯、九原万达、包百新天地、红星美凯龙等商业综合体项目，积极构建辐射带动周边城市的中高端和个性化消费中心。促进文化旅游深度融合，实施老包头记忆历史文化旅游街区项目，推动大型实景文化演出、图书类文化综合体等项目。大力发展全域旅游，加快创建土右、达茂、石拐、固阳四个全域旅游示范区。集中力量创建五当召国家5A级景区，抓好九峰山大峡谷、秦长城、希拉穆仁草原旅游区等一批旅游项目建设。实施军事文化旅游体验园、小白河文化旅游产业园等项目，培育特色旅游品牌。加强与重点客源城市和航班直飞城市的旅游合作，开发旅游精品线路，打造国内知名旅游目的地。

三是大力实施乡村振兴战略。坚持以工补农，以城带乡，走城乡融合发展的道路。深化农牧业供给侧结构性改革，促进农牧业与二三产业融合发展，落实粮食安全盟市长责任制。调整优化种养结构，积极发展特色种养殖业，加快“菜薯肉乳”主导产业转型升级，建设现代农牧业示范基地10个、都市休闲观光农牧业示范基地10个。推动现代农牧业示范园区提档升级，年内入园企业达到130家以上，3个食品加工园区新增加工企业20家以上，提高绿色农畜产品加工转化率。引导鼓励土地流转，促进农牧业规模化、集约化经营，大力培育龙头企业、合作社、专业大户、家庭农牧场等新型农牧业经营主体。支持拓展连锁直销、农超对接、物流配送、电子商务等销售途径，开展农畜产品质量安全追溯体系试点建设，着力构建现代农牧业产业体系、生产体系、经营体系。

（三）坚决打好三大“攻坚战”，筑牢经济社会发展底线。全力落实“防范化解重大风险、精准脱贫、污染防治”任务，进一步夯实决胜全面建成小康社会的基础。

一是打好防范化解重大风险攻坚战。重点防范金融风险，组建包头市地方金融监督管理局，强化属地风险处置责任，加强金融风险监测预警和防范处置，加大互联网金融监管力度，严厉打击违法违规金融活动，规范产权交易场所、小额贷款公司、融资性担保公司经营运行，坚决守住不发生区域性、系统性金融风险底线。坚持把化解政府债务作为一项重大政治任务来抓，全面加强政府债务管理，更多地运用市场化规则，盘活政府性资产，激发平台公司活力，打好预算安排、债权置换、资产重组、资源盘活组合拳，积极稳妥化解政府债务。强化预算绩效和债务限额管理，出台政府性投资项目管理办法，规范政府举债行为，严控政府债务增量，力保通过4年时间将政府债务率降至合理区间。

二是打好精准脱贫攻坚战。全面落实“六个精准”和“五个一批”要求，压实旗县区脱贫攻坚主体责任，强化部门行业扶贫及社会扶贫责任，保证现行标准下的脱贫质量，既不降低标准，也不吊高胃口。加大产业扶贫力度，依托小尾羊、草原立新、建华禽业等龙头企业，通过委托代种代养、保底收购及入股分红等方式，促进自主生产与龙头带动结合，稳定贫困户收入来源。全面落实教育、医疗扶贫措施，加快推动危房改造，让贫困户都能看得起病、上得起学、住上安全房。进一步落实政策性兜底措施，不断深化和创新社会帮扶模式，按计划完成精准脱贫任务。加大“智志双扶”力度，更加注重激发贫困人口的内生动力，建立完善防止返贫长效机制，不断巩固和提升脱贫成果。

三是打好污染防治攻坚战。全面推进中央、自治区环保督察反馈问题整改落实。持续开展大气污染防治行动，突出重点污染源专项整治，启动实施钢铁、电力等重点行业的污染深度治理工程，加强工业园区大气污染防治，全面完成煤场封闭，在工业污染源全面达标排放的基础上，进一步压减排放总量，严控排放增量。持续推动原煤散烧治理，强化露天焚烧和扬尘污染管控，严查高污染车辆，全面停止销售低于国五标准的普通柴油，坚决打赢蓝天保卫战。开展水污染综合治理，完善污水收集管网建设和污水处理厂提标改扩建，进一步提高中水利用率。强化土壤污染管控和修复，划定土地利用功能分区，建立土壤污染企业清单，强化固体废弃物和垃圾处置监管。加强农业面源污染防治和城中村、城边村环境综合整治，不断改善城乡生产生活环境。

（四）不断深化改革开放，激发各类市场主体活力。把改革开放摆在更加突出的位置，全力破解影响制约发展的体制机制障碍，拓展发展空间，厚植发展优势。

一是深入落实重点领域改革任务。进一步理顺推进改革的体制机制，增强改革方案的针对性可行性，提高改革举措的执行力落实力。深化国有资产管理体制改革，盘活资产存量，优化资产布局，促进保值增值，推动国有资本做强做优做大。深化国有企业改革，稳步推进国有企业剥离办社会职能，完成包钢“三供一业”分离移交任务。纵深推进“放管服”改革，进一步简化优化审批流程，推动“并联审批”“多图联审”，开辟园区项目审批绿色通道，不断优化营商、便民服务环境。深化商事制度改革，推行企业全程电子化登记，实现“双随机一公开”监管全覆盖。全面启动稀土新材料产业园区配售电改革。实施标准化改革，创建国家标准化综合改革试点城市。落实第二轮土地承包到期后再延长30年政策，加快完成土地草牧场确权登记颁证工作。开展经济发达镇行政管理体制改革试点，改进乡镇行政管理模式。

二是加快构建全面开放新格局。积极参与“一带一路”建设，支持包钢、一机、北重、北奔等品牌企业开拓国际市场，鼓励鹿王、丰达等企业开展产能国际合作。紧紧抓住满都拉作为呼包鄂唯一陆路口岸的有利条件，推动基础设施提档升级，建成中蒙互市贸易区一期和国家进口肉类指定口岸，推进满都拉至蒙古国赛音山达、塔奔陶乐盖2条公路项目，加快形成综合运输成本优势，大幅提升口岸竞争力，力争过货量突破300万吨。加快传化公路港和蒙西国际物流配送中心项目建设，发挥B型保税物流中心作用，完善曹妃甸“无水港”“一站式”服务，改造升级天津“无水港”，推动中亚国际班列市场化运行。创新与俄蒙合作机制，做好赴蒙棚改援建项目，以开放促发展。

三是主动开展区域合作。积极参与呼包鄂协同发展，发挥比较优势，抢占发展先机，在工业、金融、文化、旅游、商贸等领域谋划和实施一批重点项目，打造呼包鄂区域的经济辐射区和产业集聚区。积极推动包茂高速包头至东胜改扩建等基础设施互联互通工程，优化区域资源配置。加强协同创新，与呼鄂两市开展产学研合作，积极创建新型研发机构，提升科技成果转化效率。推动医疗社保、教育人才、住房保障、林业生态等资源的共建共享，强化三市环境污染联防联治，着力开创协同发展新局面。主动融入京津冀和呼包银榆经济区，加强与长三角、珠三角合作，拓展经济成长新空间。

（五）着力优化城乡环境，构建城乡协调发展格局。尊重和顺应城市发展规律，统筹规划、建设、管理三大环节和生产、生活、生态三大布局，全面增强城乡发展的系统性和宜居性。

一是着力完善城市功能。加强城市发展战略研究，启动编制新一轮城市总体规划（2021—2035），推动“多规合一”，绘制城乡统筹发展“一张蓝图”，划定“三条红线”，用红线管控城市、管控土地。开展城市设计和城市“双修”试点，注重城市风貌塑造和单体建筑设计，更多体现包头地域特色和时代特征。加快新都市区和北梁腾空区建设，实施地下管廊、海绵城市、排水防涝、污水处理、充电桩、停车场等一批基础设施工程，新改续建道路49条115公里，提升城市承载力。合理把控土地供应节奏，精准推进商品非住房去库存，加快建立多主体供应、多渠道保障、租购并举的住房制度，改造棚户区10896户，促进房地产市场健康发展。出台物业管理规定，召开全市物业管理大会，实施住宅小区物业管理提档升级三年行动，年内实现146个小区物业管理达标。加强装配式钢结构建筑发展应用，加大绿色建筑推广力度，全面提升建筑工程质量安全水平，加快推动建筑产业转型升级。推动国家生态园林城市创建，进一步提升赛汗塔拉、奥林匹克公园“城市绿肺”功能，实施昆河、二道沙河、东河生态修复治理，打造一批特色鲜明的社区公园和中小型绿地，全面构建“300米见绿、500米见园”的城市绿化格局，让绿色成为包头的城市品牌。合理配置医院、商圈等区域周边的停车设施，方便群众出行。实施城市精细化管理，推进市政公用、市容环卫、园林绿化和城市管理执法等职能协作共融，加密城市管理网格，持续开展私搭乱建、破墙开店、张贴小广告等专项整治，提高城市管理和服务水平。

二是推进县域协调发展。优化外五旗县区资源配置，加大基础设施投入、生产要素集聚和资金政策倾斜力度。推动石拐加速融入主城区，做强旅游业、大数据和现代物流产业。支持土右发展清洁能源、新型化工和新材料等主导产业，打造新的经济增长极。发挥白云、达茂区位优势，大力发展边境经济，推动稀土资源综合利用、风光新能源、现代煤化工等产业发展。帮扶固阳巩固扩大脱贫攻坚成果，建设全市重要的产业承接基地，发展特色冶金、新能源产业及旱作绿色农业。规划建设一批产业定位明确、文化内涵深厚、景观风貌独特的特色小镇，培育壮大县域经济。开展农村牧区人居环境整治三年行动，建立完善长效管理机制，加强农村牧区基础设施建设，建好管好护好运营好农村公路，推进“厕所革命”，强化垃圾污水治理，提升村容村貌，创建50个市级美丽乡村示范村。

三是加强生态文明建设。启动大规模国土绿化行动，培育一批专门从事生态保护修复的专业化企业，引导鼓励社会多方投资参与生态建设。实施新一轮退耕还林还草、京津风沙源治理和天然林保护等国家林业重点工程，扎实推进公路、村屯、厂矿园区、城镇周边、黄河流域及大青山南坡等重点区域绿化，森林覆盖率提高到17.8%。实施湿地保护恢复工程，建设包头黄河国家湿地公园，增强湿地生态功能。落实草原生态补奖政策，持续实施围封禁牧、退牧还草，积极开展草原荒漠化、沙化治理，确保基本草原生态持续改善。全面落实河长制，稳步实施以防洪安全、水污染治理、水环境改善、水生态修复为重点的城市水生态提升综合利用工程，着力打造沿黄生态走廊。不断完善生态文明制度体系，健全自然资源资产产权制度，严格执行污染物排放许可制度，探索建立市场化、多元化生态补偿机制，改革生态环境监管体制。促进低碳绿色发展，强化能源消耗总量和强度“双控”，启动智慧能源监管平台建设，实现工业能源消耗的动态监控和数字化管理。推进传统产业和工业园区循环化改造，完善再生资源回收体系，抓好国家“城市矿产”示范基地建设，创建可再生能源综合应用示范区。

（六）着力保障改善民生，促进社会和谐稳定。牢牢抓住人民群众最关心最直接最现实的利益问题，周密谋划、用心操作，尽力而为、量力而行，实实在在办好民生实事。

一是千方百计促进就业增收。坚持就业优先战略和积极就业政策，扎实做好以大学生、农牧民和就业困难人员为重点群体的就业创业工作，完善覆盖城乡的技能培训制度，确保全年新增城镇就业4万人以上，农牧民转移就业23万人以上。推进创业园孵化基地提档升级，落实好创业担保贷款等各类支持政策。健全科学合理的企业工资正常增长机制，完善机关事业单位津贴补贴和绩效工资制度，不断提高居民劳动收入和财产性收入。严格落实惠农惠牧政策，支持和鼓励农牧民就业创业，拓宽农牧民增收渠道。全面治理拖欠农牧民工工资问题，形成市场主体自律、政府依法监管、社会协同监督、司法联动惩处的工作体系，切实维护好农牧民工合法权益。

二是编密织牢社会保障网。全面实施全民参保计划，针对重点群体开展精准扩面，及时调整养老、失业等各项社会保险待遇水平。深化医保支付方式改革，严格医保资金使用管理，推行总额控费和单病种付费方式，控制医疗费用不合理增长。统筹城乡社会救助体系，推进低保制度和社会救助、社会福利、慈善事业、优抚安置等相衔接，稳步提高特殊困难群体保障标准。加快民政福利园区建设，健全完善关心关爱弱势群体工作机制，使鳏寡孤独者得到关爱。推进残疾人事业发展，建成残疾人康复中心。大力发展养老事业和养老产业，加强日间照料中心、养老服务站、互助幸福院、综合养护院以及医养服务机构等养老设施建设，打造城市养老服务示范基地。

三是促进教育质量提升和均衡发展。全面启动教育提升三年行动计划，实施校长领航工程，建立分层培训机制，提升校长办学治校能力，着力解决好中小学生课外负担重、大班额、乱办班乱补课等突出问题。实施学前教育普惠工程，积极推进23所中小学幼儿园新改扩建项目，确保公办和普惠性民办幼儿园覆盖率达到82%。实施义务教育扩优工程，推进城区强校带弱校的学区化、集团化办学，深入开展城乡学校结对共建，促进城乡教育一体化发展，努力让孩子们都能享有公平而有质量的教育。实施普通高中教育质量提升工程，推进包一中迁建等项目，建设9个普通高中学科（项目）基地，提升普通高中毕业生的优质升学率。积极支持高等院校内涵式发展，完善职业教育和培训体系，深化产教融合、校企合作，切实把高等院校打造成为集聚和培养人才的摇篮。统筹抓好民族教育特色创建、特殊教育发展、社会实践教育创新工程，鼓励社会力量兴办教育，完善教育投资多元化体制，不断满足教育资源供给多样化需求。

四是推动文化事业繁荣发展。加快公共文化服务体系建设，加大向社会力量购买公共文化服务力度，提升公共文化服务标准化、均等化水平。办好鹿城文化艺术节、交响音乐季等群众性文化活动，创作排演《双翼神马》等一批文艺精品，不断扩大“包头故事”影响力、感染力。制定加快乌兰牧骑事业发展的意见，更好地支持乌兰牧骑在新时代实现新发展，努力让广大农牧民精神生活更加丰富多彩。加强文物保护利用，实施秦长城、五当召、北梁老街巷等一批古遗址、古建筑、古民居保护利用工程，加大工业遗产保护开发力度，促进中华传统文化和地区特色文化有效传承。大力实施公民道德建设工程，不断深化道德之城、诚信之城、志愿之城建设，巩固扩大全国文明城市创建成果。

五是全力提升全民健康水平。全面开展健康城市创建工作，加快实施23项健康行动和10项健康产业工程。优化医疗布局，推进市儿童医院、包医国际医院、市蒙中医院等项目建设，构建优质高效的医疗卫生服务体系。加快医药卫生体制改革，建立现代医院管理制度，健全完善药品供应保障体系，推动家庭医生签约服务和医联体建设，努力让群众看得上病、看得起病，享受到更便捷、更优质的医疗服务。建设全民健康信息平台，依托医疗院士工作站和博士工作站，培育壮大学科和人才队伍。支持社会办医，吸引社会资金进入养老、医疗领域。坚持中西医并重，持续推动蒙中医药事业健康发展。推进平安医院建设，关心爱护医务人员身心健康，营造全社会尊医重卫的良好风气。大力实施食品安全工程，积极创建国家食品安全示范城市，加大监管力度，让群众吃得放心。落实全民健身国家战略，促进体育事业与体育产业协同发展，加强城乡体育设施建设，丰富群众健身活动，让运动休闲成为市民生活新时尚。

六是加强和创新社会治理。强化安全生产责任制，大力开展企业主体责任落实标准化、行业监管常态化、执法监察规范化、属地监管网格化建设，坚决遏制重特大安全事故。加大源头预防和矛盾纠纷排查化解工作力度，积极推动信访积案化解，加快信访法治化建设步伐，依法依规解决群众合理诉求。深入推进“平安包头”建设，进一步完善立体化社会治安防控体系和“三见警”常态化巡逻机制，持续加大缉枪治爆和打击黄赌毒、黑拐骗工作力度，不断提升人民群众安全感。坚持共建共治共享，完善社区治理体系，为群众解难题、办实事，把社区建设成为人民群众的幸福家园。加快公共法律服务体系建设，满足群众法律服务需求。认真落实党的民族宗教政策，促进民族团结、宗教和顺。支持军队改革和国防建设，落实优抚安置政策，争创全国双拥模范城“九连冠”。

（七）全面加强政府自身建设，不断提高施政能力和服务水平。各级政府及其工作人员必须牢固树立“四个意识”，自觉维护以习近平同志为核心的党中央权威和集中统一领导，始终保持为民务实清廉，增强工作本领，切实肩负起新时代的历史责任。

一要恪守为民之责。坚持密切联系群众，畅通民意表达渠道，坚持开门决策，多方汇集民智，真正使政府决策建立在广泛的民意基础上。把万家忧乐放在心头，千方百计解决事关群众切身利益的教育、医疗等热点难点问题。进一步提升政风行风热线等便民平台服务质量，完善诉求办理和督查督办机制，积极回应社会关切。树牢“功成不必在我”的理念，多做打基础、利长远的工作，多做惠民生、得民心的实事，久久为功践行为民服务的宗旨。

二要严格依法行政。认真落实市人大及其常委会的决议和决定，积极支持政协通过协商民主广泛深入参政议政，主动听取民主党派、人民团体和社会各界意见建议，坚持重大事项报告和通报制度，进一步提高人大代表建议和政协委员提案办理质量，自觉接受市人大法律监督、工作监督和政协民主监督。支持监察体制改革试点工作，加快实现对公职人员行使公权力的全面约束和统一监督。坚持“法定职责必须为，法无授权不可为”，自觉运用法治思维和法治方式推动工作。推进依法决策、民主决策、科学决策，完善市政府工作规则和市政府党组“三重一大”议事制度，建立决策后的绩效评估和责任追究制度。严格规范公正文明执法，加强行政执法规范化、信息化，不断强化法治政府建设。

三要强化实干担当。各级政府要带头讲担当、讲诚信，坚决兑现作出的承诺，以政府的担当诚信带动全社会实干有为、诚实守信。进一步强化执行力、落实力，强化督查问责，严厉整肃庸政懒政怠政行为，决不允许占着位子不干事。健全激励机制和容错纠错机制，给干事者鼓劲，为担当者撑腰，让广大干部愿干事、敢干事、能干成事。加强政府职业能力建设，大兴调查研究之风，不断增强政府公职人员学习本领、改革创新本领、科学发展本领、群众工作本领、狠抓落实本领、驾驭风险本领，确保中央、自治区和市委决策部署落实到位。

四要坚持廉洁从政。认真落实全面从严治党要求，全力抓好党风廉政建设和“两个责任”落实。强化重点领域、关键岗位监督，规范权力运行，全方位扎紧制度笼子。严格落实中央八项规定实施细则和自治区、市委配套规定，全面落实市委开展“作风建设年”的部署要求，持之以恒转作风，严防“四风”反弹回潮和隐形变异。认真开展“不忘初心、牢记使命”主题教育，严肃党内政治生活，强化党内监督，从严管理政府公职人员。严控“三公”经费支出，压缩行政运行成本，用政府的“紧日子”换取群众的“好日子”。

各位代表，历史在传承，时代在召唤。回首包头的发展历程，第一代开拓者艰苦创业，在一片荒芜的原野上建起一座体系完整的工业城市；改革开放以来，全市人民锐意进取，把一座工业城市建成了具有现代气息的文明城市；今天，新时代的历史使命落在了我们肩上，接好事业传承的接力棒，回应人民群众的新期盼，需要我们只争朝夕，担当有为。让我们更加紧密地团结在以习近平同志为核心的党中央周围，在市委的坚强领导下，以时不我待的紧迫感、舍我其谁的责任感，凝心聚力、埋头苦干，为决胜全面建成小康社会、开启全面建设社会主义现代化新征程、奋力谱写“建设亮丽内蒙古，共圆伟大中国梦”包头篇章而努力奋斗！
